# Meeting report of the fifth annual workshop on Principles and Techniques for Improving Preclinical to Clinical Translation in Alzheimer's Disease Research

**DOI:** 10.1002/alz.13742

**Published:** 2024-02-24

**Authors:** Michael Sasner, Kristen D. Onos, Paul R. Territo, Stacey J. Sukoff Rizzo

**Affiliations:** ^1^ The Jackson Laboratory Bar Harbor Maine USA; ^2^ Indiana University School of Medicine Department of Medicine Division of Clinical Pharmacology Indianapolis Indiana USA; ^3^ Indiana University School of Medicine Stark Neurosciences Research Institute Indianapolis Indiana USA; ^4^ University of Pittsburgh School of Medicine – Aging Institute Pittsburgh Pennsylvania USA

**Keywords:** Alzheimer's disease, best practices, mouse models, preclinical translation, rigor and reproducibility, training

## Abstract

The fifth annual workshop on Principles and Techniques for Improving Preclinical Translation of Alzheimer's Disease Research was held in May 2023 at The Jackson Laboratory in Bar Harbor, Maine, USA. The workshop was established in 2018 to address training gaps in preclinical translational studies for Alzheimer's disease (AD). In addition to providing fundamental knowledge and hands‐on skills essential for executing rigorous in vivo studies that are designed to facilitate translation, each year the workshop aims to provide insight on state‐of‐the‐field technological advances and new resources including novel animal models, publicly available datasets, novel biomarkers, and new medical imaging tracers. This innovative and comprehensive workshop continues to deliver training for the greater AD research community in order to provide investigators and trainees with the knowledge and skillsets essential for enabling improved preclinical to clinical translation and accelerate the process of advancing safe and effective therapeutic interventions for AD.

**Highlights:**

- Translational research is not typically available as a course of study at academic institutions, yet there are fundamental skillsets and knowledge required to enable successful translation from preclinical experiments to clinical efficacy.
- It is important that there are resources and opportunities available to researchers planning preclinical translational experiments.
- Here we present proceedings from the fifth annual NIA‐sponsored workshop focused on enabling improved preclinical to clinical translation for Alzheimer's disease research that includes didactic lectures on state‐of‐the‐field approaches and hands‐on practicums for acquiring essential translational laboratory techniques.

## 1 INTRODUCTION

The number of Alzheimer's disease (AD) cases continues to rise, and the urgency to develop effective new therapies beyond the current US Food and Drug Administration (FDA)‐approved interventions remains.^1^ As novel interventions are being developed and proposed as potential therapeutics for the treatment of AD, there are critical experiments that are necessary to inform the potential efficacy and safety prior to moving into clinical trials in AD patients. In these translational experiments, it is essential that the highest level of rigor be applied and maintained to provide assurances for successful translation from animal models to human patients. Unfortunately, there have been an overwhelming number of preclinical drug studies for which there is evidence of deficiencies in both experimental design and execution, including a lack of appropriate control groups, underpowered samples, and biased results.^2^, ^3^, ^4^ Animal models continue to be essential to the drug discovery and development process, such that therapeutics can be evaluated in the context of preclinical translation studies both to predict safety and potential efficacy prior to advancing into the clinic. Unfortunately, *preclinical translation* is not typically available as a course of study at academic institutions, and there are fundamental skill sets needed in addition to basic knowledge for the proficient execution of these studies. To this end, it is important that there are resources and opportunities available to researchers planning preclinical experiments as well as considering a career in translational research.

Since its inception in 2018, the annual workshop on Principles and Techniques for Improving Preclinical Translation of Alzheimer's Disease Research held at The Jackson Laboratory (JAX) in Bar Harbor, ME, has provided participants with necessary hands‐on and didactic training in fundamental skill sets required for conducting rigorous in vivo studies in AD mouse models.^3^ Throughout its 5 years, this workshop has been dynamic and responsive to the latest innovations in the field and is refreshed annually. In this fifth year of the workshop, the course content was expanded to include information on new animal models being generated by the Model Organism for the Evaluation of Late Onset AD (MODEL‐AD) consortium, new chemical probes and research tools being developed by Target Enablement to Accelerate Therapy Development for AD (TREAT‐AD) consortiums, an overview of available drug screening resources by the Preclinical Testing Core (PTC) of MODEL‐AD, new translational approaches including the rapidly evolving field of blood‐based and imaging‐based biomarkers and multi‐omic analyses, and content emphasizing diversity, equity, and inclusion in AD research. Faculty are specifically selected each year of the workshop based on their expertise in the relevant topics—including drug testing for AD, neuropharmacology, drug discovery and development, pharmacokinetics (PK), pharmacodynamics (PD), PK/PD modeling, in vivo target engagement, biomarkers, neuroimaging, AD genetics and genomics, mouse models, preclinical statistics, and research on diverse populations—as well as for their advocacy for rigor and reproducibility, and their personal commitment to diversity and inclusion. To expand the impact of this course, lectures were broadcast to a virtual audience, and all materials including standard operating procedures (SOPs) were made available to all participants while encouraging them to share with colleagues; however, hands‐on practicums and town hall‐style forum discussions were limited to in‐person participants.

The ultimate goal of these workshops is to improve rigor, reproducibility, and translation to the clinic as a means to accelerate the pace of advancing new treatments to patients. We believe that the content, skills, and practices addressed in this workshop are extremely important information for all researchers involved in preclinical AD studies, current and future. Thus, as part of the dissemination plan, the proceedings and lessons learned from this workshop are summarized herein.

### 1.1 Selecting the model specific for the research question

**FIGURE 1:**
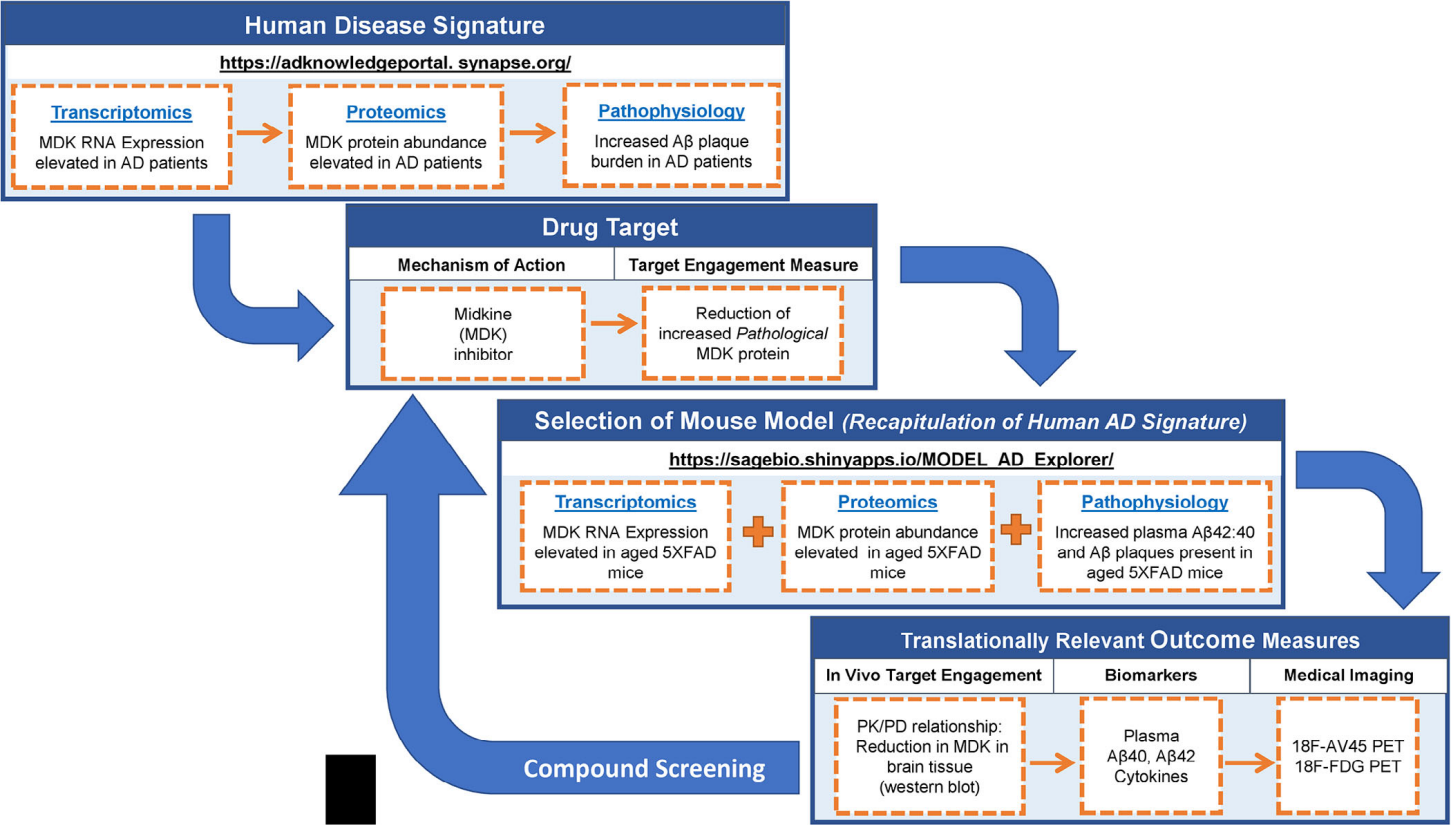
Precision translational medicine approach used by the Model Organism for the Evaluation of Late Onset Alzheimer's Disease (MODEL‐AD) consortium's Preclinical Testing Core and the Target Enablement to Accelerate Therapy Development for AD (TREAT‐AD) consortium for selecting the most appropriate mouse model matched for a drug's mechanism of action. Midkine (MDK) is an example of a target being pursued for the treatment of AD with small molecule inhibitors. Mouse models are selected based on recapitulation of human AD disease signatures at both the molecular (mult‐omic) level based on transcriptomic and proteomic data, and based on presence of AD‐related pathology and biomarkers (fluid, imaging). Exploration of the AD Knowledge Portal and MODEL‐AD Explorer data resources identified aged 5XFAD mice as a model that recapitulates MDK signatures in AD patients.^11^, ^14^ Aβ, amyloid beta; PD, pharmacodynamics; PK, pharmacokinetics; PET, positron emission tomography.

Anecdotally, a common altruistic practice among researchers is the sharing of reagents as a way to minimize costs, which also includes the sharing of mouse models, especially with research laboratories in proximity to each other within institutions. While there is certainly the benefit of minimizing the need for separate colonies, one of the major constraints of this practice is that it is essentially *model selection by opportunity*, as opposed to precisely selecting a model specific to the research question. Relatedly, many of the most widely used mouse models are based on the genetics of familial early‐onset AD (EOAD) where transgenic approaches were used to over‐express human sequences carrying mutations in amyloid precursor protein (APP) and/or presenilin (PSEN1, PSEN2), which represent only about 5% of AD patients.^5^, ^6^ Although these models show hallmark amyloid pathology, this generally occurs in young mice and therefore does not recapitulate the biological construct of aging which is the strongest risk factor for AD, and critically, tau pathology and disease‐relevant neurodegeneration are largely absent.^5^, ^6^ While tau pathology has commonly been induced in mice using transgenic overexpression of specific isoforms of human MAPT, and these models have been useful to understand the underlying biology, they do not faithfully recapitulate the more complex and age‐dependent common form of late onset AD (LOAD).^7^ In a series of presentations, Drs. Michael Sasner, Gregory Carter, and Gareth Howell from JAX describe the development and characterization of the newest mouse models being generated by MODEL‐AD including by teams from Indiana University, JAX, and The University of Pittsburgh (IU/JAX/PITT) MODEL‐AD center and the University of California Irvine (UCI) MODEL‐AD center.^7^ To date, MODEL‐AD has generated over 50 new mouse models targeting specific risk alleles that confer LOAD in patients, and that are available without licensing or intellectual property (IP) barriers to the greater AD research community from JAX.^7^ Each model undergoes a rigorous and comprehensive phenotypic characterization using standardized best practices with an emphasis on rigor and reproducibility. The comprehensive characterization pipelines include aging time points up to 24 months, are powered to detect effects of sex, and prioritize translational outcome measures including gene expression and proteomic profiling, fluid biomarkers, medical imaging, and histopathology. The extensive library and variety of mouse models and their comprehensive phenotypic characterization data provide an opportunity for the selection of a mouse model specific to the research question being proposed. All of these data including the available models and characterization data are accessible to researchers via the AD Knowledge Portal, a National Institute on Aging (NIA)‐funded open‐access data repository for AD research.^8^ Related to this, Drs. Paul Territo of Indiana University School of Medicine and Stacey Rizzo of The University of Pittsburgh School of Medicine described the MODEL‐AD PTC drug screening pipeline resource, which uses a precision medicine approach that leverages the extensive characterization data from the MODEL‐AD models to select and match the specific mechanism of action of a compound being interrogated in the pipeline to the most appropriate model.^9^ Rather than using the “selection by opportunity” mouse model approach, the PTC selects the most appropriate model to enable translation from mouse to human for in vivo target engagement and PK/PD relationships^9^ (Figure 1). For example, in simple terms, if the compound is directly targeting tau protein and there is no disease‐related tau pathophysiology expressed in the mouse model to target, then it is unlikely a preclinical translational study will be successful in that model system. Figure 1 illustrates this approach using the example of compounds targeting pathological levels of midkine in the brain, for which small molecule inhibitors have been pursued as potential therapeutic interventions for AD, and which is also a target nominated by the Accelerating Medicine Partnership for AD (AMP‐AD) consortium.^9^, ^10^, ^11^

At the conclusion of these lectures, a breakout session led by Dr. Howell challenged the participants to put their newly learned knowledge of the available mouse model resources into practice. During this exercise, participants formed teams and took the opportunity to share specific aims they were developing for their own research and select the most appropriate models to address their research questions.

### 1.2 Evolving technologies for advancing AD research

**FIGURE 2:**
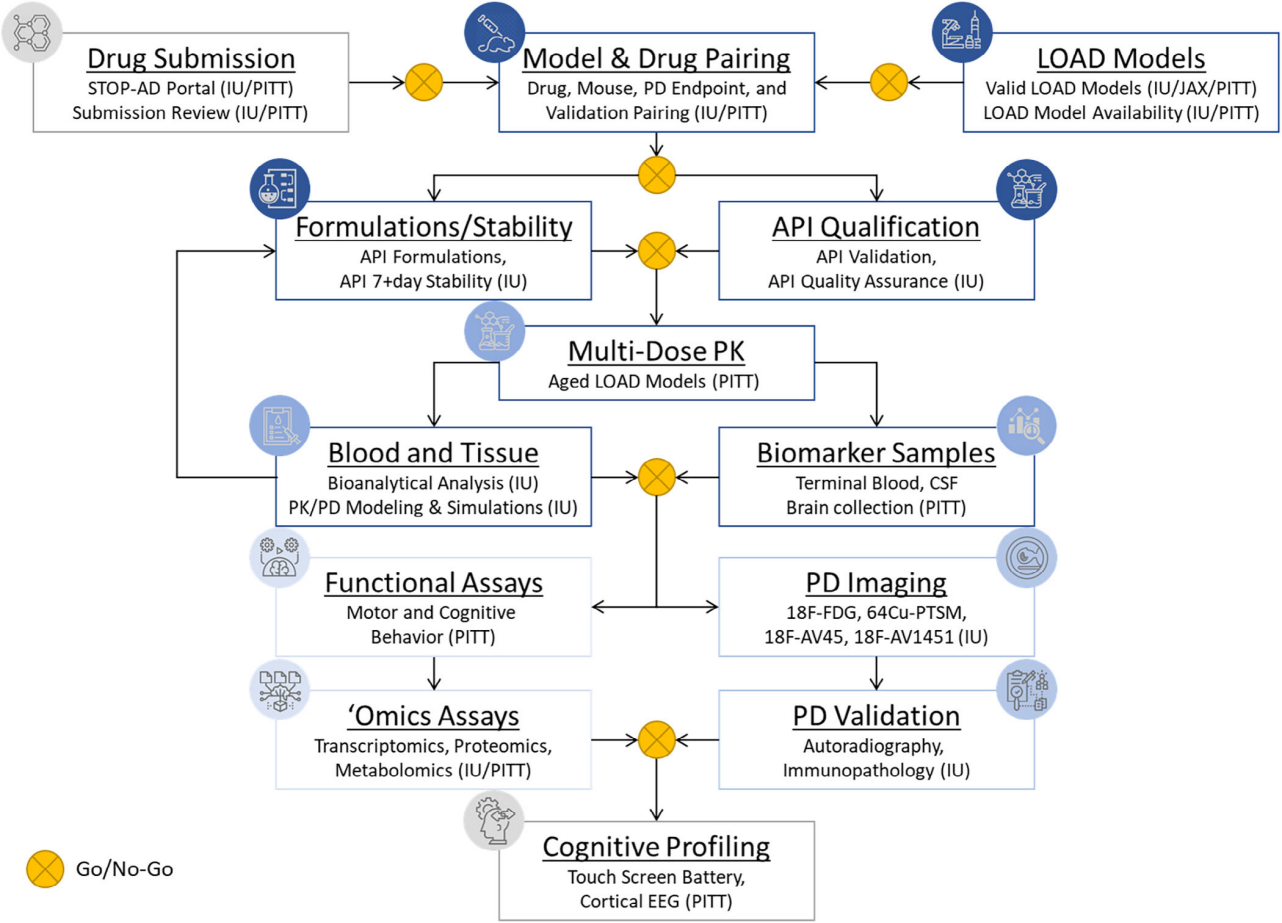
The Model Organism for the Evaluation of Late Onset Alzheimer's Disease (MODEL‐AD) consortium's Preclinical Testing Core drug screening pipeline is a resource available to the greater research community with a priori go/no‐go decision points that prioritize translational outcomes to permit unbiased rigorous drug screening. API, active pharmaceutical ingredient; CSF, cerebrospinal fluid; EEG, electroencephalography; IU, Indiana University; JAX, The Jackson Laboratory; LOAD, late onset Alzheimer's disease; PD, pharmacodynamics; PITT, University of Pittsburgh; PK, pharmacokinetics; STOP‐AD, Selecting The Optimal Pharmaceutical for preclinical drug testing in AD.

The field of biomarkers for AD is a rapidly evolving area and the challenges and opportunities were highlighted by Dr. Jeff Dage from Indiana University School of Medicine.^12^ Dr. Dage shared an overview of the drug discovery process, the types of biomarkers, and how biomarkers are used in the drug development process including for tracking PD changes. He also emphasized the importance of developing and validating biomarkers early in the drug discovery process, well before clinical trials, given the lengthy validation studies required in animal models. Importantly, Dr. Dage highlighted how the diagnosis of AD is now being facilitated by biomarkers which will enable earlier detection and intervention^13^ (Figure 2).

Dr. Nicholas Seyfried from Emory University presented the most recent advances on multi‐omics analysis for AD including specific details on how proteomic approaches are being used to identify new drug targets. Dr. Seyfried described the work from the AMP‐AD consortium that analyzed over 2000 human brain tissues by mass spectrometry‐based proteomics and identified consensus AD brain protein co‐expression networks.^14^ Technical details explaining the differences in experimental approaches including label‐free quantification (LFQ) versus tandem mass tag (TMT) were also shared as well as methods for data analysis and interpretation. Dr. Seyfried also highlighted how emerging proteomic data from mouse models developed by MODEL‐AD are recapitulating many of the human AD protein modules. Finally, he described how proteomics is being used both to identify novel drug targets and as biomarkers that can be tracked longitudinally in cerebrospinal fluid (CSF) in both animal models for translational studies and ultimately in humans to track disease trajectory as a surrogate for brain tissue^15^, ^16^, ^17^ (Figure 1).

Dr. Pascal Sanchez from Denali Therapeutics presented an in‐depth overview of his approach to the preclinical testing process, with specific real‐world examples. He presented a typical decision‐making matrix with potential outcomes (eg, target engagement, change in a relevant pathway biomarker), and emphasized the need for criteria for go/no‐go decisions that have been established a priori. He explained strategies for target prioritization and emphasized a framework to increase translational predictability of AD models, with a focus on more proximal readouts (eg, phosphorylation of a target) instead of more distal, less well‐conserved modalities such as behaviors.^18^ Specific vignettes discussed included the development of a novel knock‐in model,^19^ a strategy to deliver a therapeutic across the blood‐brain barrier,^20^ and the development of a Trem2‐activating antibody as a potential therapeutic.^21^

### 1.3 Hands‐on practicums

The basic fundamentals of designing, executing, analyzing, and interpreting data from in vivo PK experiments is essential to the drug discovery process and indispensable for enabling preclinical to clinical translation. As highlighted in a lecture by Dr. Shreaya Chakroborty from the NIA, curated data from more than 1200 preclinical studies of potential AD therapeutics in animal models lacked inclusion of PK data or adequate exposure levels of many compounds that ultimately failed in clinical trials.^2^, ^3^, ^4^ As part of the MODEL‐AD PTC and the TREAT‐AD centers, in vivo PK is used to prioritize or deprioritize compounds and is an early go/no‐go gate for compound screening that requires minimal resources but maximizes predictive in vitro and in silico data^9^ (Figure 2). Therefore, following a required Animal Welfare orientation and training practicum, participants were approved by the internal animal care and use committee (IACUC) at JAX to conduct approved procedures on mice. The multiple hands‐on sessions included training on procedures for dosing including oral gavage, serial blood collections using established protocols for in vivo PK sampling techniques, methods for CSF sampling and terminal tissue collections, and basic practices for following the ARRIVE guidelines^22^ including methods for blinding, randomization, and counterbalancing of treatment groups. This training was put into practice during a mock in vivo PK study that emphasized timing of sample collections, accuracy and precision, and documentation. At the completion of the mock in vivo study, Dr. Sara Quinney from Indiana University detailed methods for sample analysis from in vivo PK experiments and the importance of PK/PD modeling. Drs. Quinney and Territo provided participants with a Microsoft Excel workbook to enable interpretation of bioanalytical results including calculating PK parameters (eg, T_1/2_, C_max_, AUC, CL/F, Vd/F, etc.). Together the hands‐on components of this workshop provide participants with the tools to execute and interpret important PK data to confirm that the compounds they pursue in their own laboratories have appreciable exposure levels and identify optimal dosing regimens (eg, dose frequency, dose range) for their studies in their own laboratories.

Finally, Dr. Vivek Philip from JAX provided a statistical practicum which included methods for conducting power analyses which should be calculated specific to the model and assays being performed. In addition, an overview and guidance on the selection of appropriate statistical tests which are dataset dependent was also discussed. Dr. Philip also emphasized bias in analysis methods including *p*‐hacking and executing simple *t*‐tests which are not appropriate for complex datasets beyond comparing two groups.^23^ This session provided the opportunity for participants to get assistance with experimental designs for their upcoming studies in their respective laboratories.

### 1.4 Importance of diversity in AD research

AD research would be strengthened significantly by increasing diversity. This not only includes the recruitment and active engagement of participants who have historically been underrepresented, but also extends to the perspectives and experiences from diverse researchers as well as the incorporation of aspects of genetic diversity into research models that are used to study AD.

Dr. Lisa Barnes from Rush University opened this session by providing an overview of the challenges we are facing as a field by historically focusing on White populations for the study of disease trajectories and as cohorts for therapeutic studies. Dr. Barnes emphasized that AD is one of the most feared diseases of old age by everyone, regardless of ethnicity, race, or ancestral origin. US census data indicate that the demographics of the US population are expected to shift from what has historically been a majority White population to a majority population for people of color.^24^ These data, coupled with research showing that African Americans are two times more likely than White Americans to have AD,^25^ demonstrate the clear and pressing need to include people of color in AD research and clinical trials, inclusive of Black Latino, Asian, American Indian, and Alaskan native groups. Dr. Barnes highlighted the challenges of current standard recruitment practices as directly contributing to the disparity in AD research. For example, most AD studies recruit from well‐resourced clinics in academic medical centers leading to selection bias (small levels of diversity, highly educated and insured, etc.), and with inclusion/exclusion criteria favoring White participants (eg, few chronic co‐morbidities, access to transportation). In fact, a recent report determined that the largest data‐producing studies for AD had an overwhelmingly White/Caucasian bias.^26^ Furthermore, minorities are consistently underrepresented in clinical trials which limits the ability to conclude efficacy or assess safety in non‐White populations.^27^, ^28^ In fact the recently FDA‐approved drug aducanumab included less than 2% of Black patients in clinical trials, with similar issues of limited diversity for people of color representing only 20% and 10% respectively, in trials of lecanemab and donanemab.^28^, ^29^ In order to address these and other disparities, Dr. Barnes and colleagues established the Minority Aging Research Study (MARS), aimed at building an infrastructure and resource for investigators interested in understanding the aging profile of older African Americans.^30^, ^31^ Recruitment processes for MARS are a paradigm shift from traditional approaches that require clinic visits; instead, they engage individuals at community events such as church‐related activities and senior centers. Overall, this work continues to make great strides in understanding how social determinants influence brain health including direct links between discrimination and inflammation, along with higher burdens of vascular conditions such as hypertension and diabetes that vary across populations of AD patients and will be important for understanding how best to treat individuals that are non‐Caucasian.^30^, ^31^, ^32^, ^33^, ^34^

Dr. Kristen Onos from JAX highlighted research approaches aimed at addressing the “diversity gap” in genetic studies through the inclusion of participants from previously underrepresented ethnicities into the Alzheimer's Disease Sequencing Project (ADSP) Diversity Initiative.^35^ Dr. Onos addressed criticisms faced by researchers for failures of historical AD mouse models to translate preclinical data from drug studies into clinical efficacy, and highlighted the opportunity of new genetically diverse mouse models that have the potential to model the “subtype heterogeneity” in AD patients.^36^ In order to provide the trainees with context of how mouse models have been established for AD research, Dr. Onos provided a brief history on the C57BL/6 (B6) strain including its use as a background strain for biomedical research as well as for research in AD. The B6 mouse strain was the result of random crosses between three major subspecies of the house mouse: *Mus Musculus domesticus, Mus Musculus musculus*, and *Mus Musculus casteous*, that were collected and kept as pets by people known as mouse fanciers in the 1800s.^37^, ^38^ Thus, the B6 mouse was created by chance and not by targeting of any specific genes or physiologic traits related to human health. The mouse strain was eventually shared with researchers at Harvard in the early 1900s, which ultimately led to its wide propagation and use in research.^37^, ^38^ Dr. Onos described three main approaches for integrating genetic diversity in mouse models for AD research: first, through assessments being performed in more than one genetic background/mouse strain; second, by using mapping crosses through two strains such as F1 or recombinant inbred lines; and third, through the use of multi‐strain mapping populations such as the Collaborative Cross (CC) and Diversity Outbred (DO) lines. She provided examples of each of these types of studies and pointed out the pros and cons of each approach dependent upon the specific research question being addressed. She introduced what are known as wild‐derived mouse strains which comprise the founders of the CC and DO mouse populations, and how AD researchers are incorporating these lines into their research to model genetic and phenotypic diversity.^39^, ^40^ Dr. Onos highlighted the utility of the wild‐derived mouse strains for immune system functioning given that they were caught more recently in the wild from different regions across the world and while they are maintained as inbred, the critical processes of immune system functioning remain relatively conserved.^39^, ^41^ Course participants were enthusiastic about learning these approaches, and previous participants have gone on to use these strains in their research.^42^, ^43^

### 1.5 Expanding resources for supporting translational preclinical studies of AD

Dr. Suzana Petanceska of the NIA provided an overview of the NIA AD/AD and related dementias (ADRD) Translational Research Program for Accelerating Therapy Development for Alzheimer's Disease through Open Science. The overview included a history and trajectory of the NIA's resources and infrastructure to support AD research and highlighted a number of consortia that are part of the Open Science Programs Enabling a Precision Medicine Approach to Drug Development for AD. These include, in addition to MODEL‐AD and AMP‐AD, Resilience‐AD, Molecular Mechanisms of Vascular and Metabolic Factors in AD (M2OVE‐AD), molecular mechanisms of neuropsychiatric symptoms of AD (Psych‐AD), and TREAT‐AD. Dr. Allan Palkowitz from Indiana University School of Medicine provided an overview of the two TREAT‐AD Centers including the Emory/Sage/Structural Genomics Consortium center and the Indiana University‐Purdue University Center. The TREAT‐AD centers were established in 2019 in response to the National Institutes of Health's funding opportunity announcement RFA‐AG‐19‐010, with a major objective to diversify and reinvigorate the AD drug development pipeline by providing the research community with high quality target‐enabling tools to investigate new therapeutic hypotheses, leveraging rigorous and unbiased efficacy testing in AD models through the MODEL‐AD program, and with open dissemination of all data and resources.^10^, ^44^, ^45^, ^46^ Dr. Palkowitz described the pipelines for the TREAT‐AD centers and how targets identified from the AMP‐AD consortium are being pursued for drug discovery including incorporating the approaches for improving preclinical to clinical translation as established by the MODEL‐AD PTC.^9^, ^44^, ^45^, ^46^ More specifically he provided insight into the prioritization of SHIP1 (*INPP5D*) as a target by the IU‐Purdue TREAT‐AD center and shared data for the biological hypothesis, SHIP1 ligand discovery approach, lead compound characterization, and translational PD studies being pursued.^46^ Importantly, he provided information on where researchers can access TREAT‐AD resources including Target Enabling Packages (TEPs) which are accessible through the AD Knowledge Portal (https://adknowledgeportal.org) as well as the TREAT‐AD website (https://treatad.org).

To orient participants to accessing the resources of the AD Knowledge Portal, Dr. Abby Vander Linden from Sage Bionetworks led a demonstration of the portal. Through the demonstration, participants accessed data in real time from the AMP‐AD, MODEL‐AD, and TREAT‐AD Centers, as well as other programs supported by the NIA. Dr. Vander Linden provided detailed explanation of data curation and step‐by‐step methods for ease of access of the variety of data types in the portal. Participants also were introduced to the MODEL‐AD Explorer (https://modeladexplorer.org) which enables users to easily access phenotypic data on mouse models characterized by MODEL‐AD. This includes pathology data as well as comparative gene expression analysis of mouse models with human AD gene signatures. Dr. Vander Linden also provided links to R tutorials and Python commands so users can download and work with the data directly, and showed how to appropriately cite these data which can be used in researchers own publications.^47^

In addition to mouse model data that can be accessed via the MODEL‐AD explorer, Dr. Michael Sasner also presented additional resources for finding and selecting research tools and mouse models, and discussed the benefits of knock‐in mouse models compared to the traditional transgenic strains. He highlighted the AlzForum website databases for finding information on research models (https://alzforum.org) as well as oriented participants to the JAX website (https://jax.org) where they could not only select and order mouse models of interest, but also find extensive genetic and practical information, such as protocols for genotyping. He also shared information on how to understand nomenclature to clarify strain and genetic background information on the animals, which is essential to report for reproducibility.

As a resource for enabling testing of potential therapeutics for the greater AD research community, Drs. Paul Territo and Stacey Rizzo presented details on the Selecting The Optimal Pharmaceutical for preclinical drug testing in AD (STOP‐AD) portal.^48^ The MODEL‐AD PTC has established a rigorous and unbiased multi‐tiered screening strategy for evaluating compounds as potential therapeutic interventions for AD.^9^ The PTC pipeline prioritizes translational assessments of PK and PD measures including positron emission tomography (PET)/computed tomography (CT) and magnetic resonance imaging (MRI), as well as behavioral assessments and molecular profiling in response to drug treatment in mouse models validated by MODEL‐AD at minimal costs to the investigator (Figure 2). Given the intensive resources required for the comprehensive drug screening conducted by the PTC, as described by Dr. Territo, the team has developed a framework that provides an unbiased ranking of compounds nominated by the community for selection and prioritization.^48^ Researchers are encouraged to submit nominations for compounds to be screened by accessing the following portal: https://stopadportal.synapse.org.

Dr. Stacey Rizzo provided an overview of the newest addition to the NIA's Open Science programs for enabling a Precision Medicine Approach to Drug Development for AD: the Marmosets as Research Models for AD (MARMO‐AD) consortium. MARMO‐AD was established to bridge the rodent to human translational gap by enabling the study of primate‐specific mechanisms that may underlie the pathogenesis and progression of AD.^49^ MARMO‐AD is positioned to leverage knowledge from the spectrum of NIA open science consortia including MODEL‐AD, TREAT‐AD, and AMP‐AD. Dr. Rizzo shared opportunities for non‐human primate models for the study of AD and emphasized the natural genetic heterogeneity of the marmosets, their sophisticated behavioral and social repertoire, improved sequence homology of AD risk genes and proteins to human relative to rodents, and the conserved prefrontal cortical areas of the brain with human that are important for higher order cognitive functions which remain a translational challenge for rodent to human studies. Importantly, all resources from MARMO‐AD will be shared with the greater AD research community.

In addition to the data and model resources described above as part of the NIA Open Science initiatives, it is essential that researchers also understand opportunities available for grant funding and training opportunities. Dr. Stefania Fornier from the Alzheimer's Association shared a number of funding opportunities for researchers at all levels across basic and clinical science, including those new to the field, diversity initiatives, and specific opportunities for using mouse models and resources developed by MODEL‐AD (https://www.alz.org/research/for_researchers/grants/types‐of‐grants/discovery‐grant‐program). Dr. Maria Carranza from the Office of Strategic Extramural Programs at NIA shared information on a range of resources provided by the NIA including funding opportunities for grants as well as insight into support for transitioning to independence, different types of awards and grants, and training resources. Dr. Carranza emphasized that NIA program officers are available and accessible, and researchers should not hesitate to contact them to learn more about specific programs, as well as general guidance and support.

Taken together, there are significant resources and infrastructure that are available to researchers including the resources of the NIA‐funded R13 mechanism supporting this workshop. The workshop aims to provide young investigators (and investigators new to the AD field) with a guide to navigate this wealth of resources.

One unique aspect of the workshop is time set aside for “town hall” type informal discussions for faculty to share their experience in addressing any issues brought up by the students. This has included career guidance (eg, comparing academia with the pharma/biotech environment); discussions on Diversity, Equity, and Inclusion (DEI); ethical issues concerning data interpretation and animal use; learning how to manage people and teams in the lab environment; productively interacting with institutional responsibilities (committee work, HR); and both writing and reviewing grants and manuscripts.

## 2 DISCUSSION

In its fifth year, this workshop continues to provide a unique training environment and instructional strategies geared to overcoming existing training gaps in preclinical translational studies for AD. Evidence for success of this workshop include the ongoing scientific interactions and collaborations between the participants and course faculty, as well as peer‐to‐peer networks established among the participants. Many of the faculty serve as mentors for trainees and young investigators as they progress through their careers. Course evaluations provided participants with the opportunity to share feedback. Responses to the survey question regarding the most valuable learning experience from the course included the following: knowledge of the range of mouse models available for AD, definitions of biomarkers, consequences of not conducting PK studies for compounds being proposed as drugs in animal models, genetic and phenotypic differences across various C57BL/6 substrains, visualization methods on MODEL‐AD explorer, resource sharing, concepts of pharmacology and practical application, how to find and apply PK and PD to research findings, CSF collection and brain dissections, how PET/CT and MRI tools work, and how they are applied to AD phenotyping and therapeutics testing. Multiple participants have stated that they plan to share their knowledge, skills and resources (eg, SOPs) gained from the course and immediately implement them upon returning to their labs. Recent publications from previous workshop attendees also highlight the success of the approaches for improving preclinical to clinical translation, as well as the dissemination of resources including open access raw data available to the research community presented as part of this workshop.^41^, ^42^, ^43^, ^50^

This hands‐on workshop is an annual event at JAX in Bar Harbor, Maine. The course is open to trainees as well as experienced researchers and clinicians at all levels, and across academia and industry. Those new to the field and those from underrepresented backgrounds are especially encouraged to attend. Information and registration can be accessed at the JAX Courses and Conferences website: https://www.jax.org/education‐and‐learning/course‐and‐conferences/principles‐and‐techniques‐of‐alzheimers‐disease

## AUTHOR CONTRIBUTIONS

Michael Sasner, Kristen D. Onos, Paul R. Territo, and Stacey J. Sukoff Rizzo are course directors, designed the workshop content and wrote the manuscript.

## CONFLICT OF INTEREST STATEMENT

Michael Sasner and Kristen D. Onos are full‐time employees of The Jackson Laboratory. Paul R. Territo is a full‐time employee of Indiana University School of Medicine. Stacey J. Sukoff Rizzo is a full‐time employee of The University of Pittsburgh School of Medicine and has served as a consultant for Genprex, Hager Biosciences, and Sage Therapeutics. Author disclosures are available in the supporting information.

## Supporting information

Supporting Information

## Data Availability

Data sharing is not applicable to this article as no datasets were generated or analyzed during the meeting. The MODEL‐AD SOPs provided during the hands‐on workshop are available through the AD Knowledge Portal (https://adknowledgeportal.synapse.org).
